# Color Comparison Between Intraoral Scanner and Spectrophotometer Shade Matching: A Systematic Review and Meta‐Analysis

**DOI:** 10.1111/jerd.13309

**Published:** 2024-09-09

**Authors:** Viktória Vitai, Anna Németh, Brigitta Teutsch, Kata Kelemen, Alíz Fazekas, Péter Hegyi, Orsolya Németh, Beáta Kerémi, Judit Borbély

**Affiliations:** ^1^ Centre for Translational Medicine Semmelweis University Budapest Hungary; ^2^ Department of Prosthodontics Semmelweis University Budapest Hungary; ^3^ Institute of Pancreatic Diseases Semmelweis University Budapest Hungary; ^4^ Department of Radiology, Medical Imaging Centre Semmelweis University Budapest Hungary; ^5^ Institute for Translational Medicine, Medical School University of Pécs Pécs Hungary; ^6^ Faculty of Dentistry, Department of Community Dentistry Semmelweis University Budapest Hungary; ^7^ Faculty of Dentistry, Department of Restorative Dentistry and Endodontics Semmelweis University Budapest Hungary

**Keywords:** accuracy, digital dentistry, precision, repeatability, shade determination tool, tooth color, trueness

## Abstract

**Objective:**

This systematic review and meta‐analysis compared the accuracy of intraoral scanners and spectrophotometers in determining tooth shade.

**Materials and Methods:**

An electronic search of five databases (PubMed, Scopus, Embase, Web of Science, CENTRAL) was conducted on October 19, 2023. A total of 163 studies were identified from the databases, of which 23 articles were eligible for inclusion. In vivo and in vitro quasi‐experimental studies were included. After data extraction, a quantitative analysis was performed to determine the accuracy of the intraoral scanner in subgroups using four outcomes: trueness and precision with different measurement locations. A random‐effects model was used to pool effect sizes. The pooled proportion with a 95% confidence interval (CI) was used for the effect size measure.

**Results:**

Eleven articles were included in the meta‐analysis. Trueness with the intraoral scanner was between 0.28 (CI: 0.09–0.60) and 0.38 (CI: 0.24–0.53). Repeatability was between 0.81 (CI: 0.64–0.91) and 0.85 (CI: 0.74–0.92). Trueness showed low, and precision had moderate certainty of evidence.

**Conclusion:**

The trueness of shade matching with intraoral scanners is low compared to spectrophotometers, although the precision is considered high and is similar to spectrophotometers.

**Clinical Significance:**

Shade determination with intraoral scanners is not recommended.

## 1 Introduction

The past few decades have seen a sharp rise in esthetic standards. When a dental restoration is constructed, it is important to ensure that the patient is satisfied with the function and esthetic of the final restoration. Zirconia and lithium disilicate materials are some of the most popular materials. Determining the right color for the patient is typically seen as challenging in achieving the final esthetic result [1, 2].

Tooth shade can be measured in a variety of ways. Visual shade determination, among the most common approaches, has been used extensively for many years. Different shade guide systems, such as the Vita Classical (VC) shade guide system and the Vita 3D‐Master shade guide system (3D), can be utilized for the subjective process of visual shade determination [3, 4]. The Vita Toothguide 3D‐Master shade tabs are still criticized for their inability to accurately represent darker teeth shades; however, its 3D design is believed to offer superior precision compared to the VC shade guide due to scientific backing for its construction [5, 6, 7, 8]. Dentists often prefer to use a visual shade determination approach; however, the accuracy of this method depends on other elements, including the operator, illumination and the background [9, 10, 11, 12].

The market has witnessed the emergence of digital objective techniques to mitigate the adverse effects of shade matching. Tools such as digital cameras, spectrophotometers (SPs), and colorimeters have been introduced [13, 14, 15]. Despite potential variations between these devices, their precision in measuring tooth shade surpasses traditional visual methods. Consequently, they are gaining popularity among dentists due to their ability to streamline procedures and save valuable time [9, 16, 17, 18].

SPs convert the reflected light's measured amount and spectral composition into tristimulus data [14, 19]. Measurements are usually taken at a single point on the tooth surface where the light is perpendicular to the surface [20]. Because of their high repeatability, extensive research, and suitability as gold standards for studies on tooth‐color measurement, SPs are considered to be highly reliable instruments [21].

Intraoral scanners (IOSs) are used to take impressions during digital workflows. The market for IOSs is expanding, and several manufacturers are available with various models that offer special features, including tooth shade determination, caries detection, and smile design. Although not currently available for every instrument on the market, there is an increasing trend toward built‐in tooth shade determination, typically using a light‐emitting diode (LED) as the light source; the IOS takes many images from different angles to reproduce a three‐dimensional dental arch image and then using VC and 3D shade criteria, the software can ascertain the tooth shade from the generated file [20]. However, the literature has conflicting information on IOSs for shade determination in routine dentistry treatment, as their accuracy has not yet been proven properly [22].

Accuracy is defined in ISO 5725‐1 as a combination of trueness and precision. All potential random components and a common systematic error or bias component during the measurement process are combined to form the word accuracy in general. Trueness is the degree to which the true or accepted reference value and the arithmetic mean of a number of test findings match. This reference value is an indicator of systemic errors. Bias is typically used to express trueness metric [23].

Precision is the degree of agreement between independent test results achieved under specified conditions. Precision also refers to the overall variability observed between successive measurements. Repeatability and reproducibility are two specific subgroups of precision, according to variability, variables that are either fixed or changeable [23, 24].

The relationship between measurement error magnitude in observed measurements and intrinsic variability in the “true,” “error‐free,” or underlying quantity level among subjects is known as reliability. The difference between repeated measurements taken on the same subject under the same circumstances is called the repeatability of measurements. This indicates that measurements are made using the same tool or technique, by the same operator, and within a brief period, during which the underlying value can be considered constant. Reproducibility describes how measurements taken on a subject under various settings can differ.

A number of methods can be used to measure shade‐matching accuracy. Trueness and precision (repeatability and reliability) of shade determination techniques can be demonstrated by match or agreement in percentages or by expressing color differences [22].

There are several formulas for calculating color difference (ΔE); the CIEDE2000 (ΔE_00_) formula is the most recent and widely accepted one and is recommended by the International Commission on Illumination (Commission Internationale del'Eclairage, CIE) [25]. Color difference results can be translated into clinical practice using the 50:50% acceptability threshold (AT) and the 50:50% perceptibility threshold (PT). The CIEDE2000 formula yielded the following color difference thresholds: PT ΔE_00_ = 0.8 and AT ΔE_00_ = 1.8 [26].

The aim of this systematic review and meta‐analysis (MA) was to investigate accuracy, trueness, and precision (repeatability) of tooth shade selection with IOSs. If IOSs prove to be as accurate as SPs, they could serve as objective and time‐efficient tools for shade selection, seamlessly integrated into comprehensive patient case documentation.

The research hypothesis was that there was no significant difference in the accuracy of shade selection between IOSs and SPs.

1. The null hypothesis was that there was no significant difference in shade selection between IOSs and SPs when trueness was expressed in match percentages.
2. The alternative hypothesis was that the repeatability of IOSs is high with a clinically acceptable match percentage.

## 2 Methods

This systematic review and MA was based on the recommendations of the PRISMA 2020 guidelines (Data S1) [27], and followed the Cochrane Handbook [28]. The protocol of the study was registered on PROSPERO (registration number CRD42022330109) and was fully adhered to. The primary research question of the MA was, “What is the accuracy of shade selection with IOSs?”

### 2.1 Inclusion Criteria and Exclusion Criteria

PIRD elements (population, index test, reference test, diagnosis of interest) were used to identify studies: we included studies that investigated shade matching with IOSs compared to SPs. Outcomes were accuracy measured in match percentage or color difference in ΔE and repeatability measured in match percentage in VC and 3D shade guide systems.

Both in vivo and in vitro quasi‐experimental studies were included that matched our PIRD framework. Articles using shade selection on natural teeth or other specimens such as blocks, shade tabs, and crowns were included. In clinical studies where shade selection was done on patients' natural teeth, the inclusion criteria were subjects without any restorations (fillings, crowns, bridges) of the shade determination area. Discoloration and plaque, if present, should be removed. Exclusion criteria were bleaching and any restoration discoloration or caries in the area of shade selection. In the in vitro studies, shade determination with known tooth shade of the specimens was involved, and also those where a reference measured the original shade.

Review articles involving SPs or IOSs, articles about the IOS's application in the fields of orthodontics, periodontics, and maxillofacial surgery, articles about intraoral scanning accuracy, caries diagnosis, tooth wear monitoring, digital prosthesis scanning, marginal fit evaluation, virtual registration of interocclusal relations, soft tissue examination, implant surgery, aligner fabrication, maxillofacial rehabilitation, diagnosis of periodontal diseases, deep learning, and artificial intelligence were all excluded during the title and abstract selection. Preprints that did not undergo peer review and case studies were also excluded.

### 2.2 Information Sources

An electronic search was conducted via PubMed, Scopus, Embase, CENTRAL, and Web of Science databases on the May 5, 2022 and updated on the October 19, 2023. No restrictions were applied during the search. A citation chaser program (https://estech.shinyapps.io/citationchaser/) [29] was used to find additional cited or referred articles in the articles included.

### 2.3 Search Strategy

During the systematic search, the main terms in the search key were shade determination, IOS, and SP (Data S2).

### 2.4 Selection Process

The selection was performed by two independent review authors (V.V. and A.N.) after duplicates were removed, articles were screened by title and abstract. Abstracts of the records were examined to determine which studies were appropriate for full‐text review. Two independent reviewers (V.V. and A.N.) conducted the screening process. A third reviewer (J.B.) resolved any disagreements.

### 2.5 Data Collection

Two independent authors performed data extraction, and a third author resolved disagreements here. The following information was retrieved by data extraction from relevant studies: author(s), year of publication, title, location, center, period, study design (in vivo, in vitro), population, shade subjects (patients, known colors of block or shade tabs), patient demographics (age, gender, inclusion criteria, exclusion criteria), number of measurements performed at a single location, measurement locations (cervical, central, and incisal region), type and number of teeth, sample size, operators (number, experience, inter‐operator agreement), shade measurement methods, type of IOS, software version, type of SP, outcome type, and value. If data in the articles was insufficient, the authors were contacted by email to request clarification and any missing data.

### 2.6 Study Risk of Bias Assessment

For risk of bias, the assessment was performed independently by two authors (V.V. and A.N.) with the Joanna Briggs Institute (JBI) Critical Appraisal Checklist for Quasi‐Experimental Studies [30], which was also used in two other systematic reviews of the topic [22, 31]. In case of disagreement, a third author (J.B.) decided the final value. The overall risk of bias was generated from the answers to nine questions (low risk: yes, high risk: no, unclear risk: unclear), and the percentage of risk types was calculated.

### 2.7 Certainty Assessment

The certainty of evidence for each subgroup was determined using GRADE Pro [32].

### 2.8 Synthesis Methods

Subgroups were created according to different outcomes, measurement locations, and IOS types. Trueness and precision groups were generated first. If sufficient data were available, these groups were divided into subgroups with measurements in the incisal, central, cervical, or all tooth locations and also in subgroups of IOS type. As we assumed considerable between‐study heterogeneity in all cases, a random‐effects model was used to pool effect sizes. To calculate study proportions, the total number of patients and those with the event of interest were used from each study. The pooled proportion with a 95% confidence interval (CI) was used for the effect size measure. Results were considered statistically significant if *p*‐values were <0.05. We summarized the findings of the MA in forest plots. Where applicable—the study number was sufficiently large and not too heterogeneous—we also reported the prediction intervals (i.e., the expected range of effects of future studies) of results.

Additionally, between‐study heterogeneity was described by the Higgins & Thompson's I [2] statistics [33].

We planned to assume a possible small study bias if the *p*‐value was less than 10%. (However, we kept in mind that the test had limited diagnostic assessment below ~10 studies).

Potential outlier publications were explored using different influence measures and plots [34].

All statistical analyses were performed with R (R Core Team 2023, v4.3.0), using the meta (Schwarzer 2023, v6.2.1) package for basic MA calculations and plots, and diameter (Cuijpers, Furukawa, and Ebert 2022, v0.0.9000) package for additional influential analysis calculations and plots [35, 36, 37].

For pooling the effect size, we used a random intercept logistic regression model method—a more specific, random intercept logistic regression model—to pool proportions (as recommended by Schwarzer et al. and Stijnen et al. [38, 39]).

We used the Hartung–Knapp for CIs [39, 40, 41].

To estimate the heterogeneity variance measure (*τ* ^2^), we used the maximum likelihood method for the prevalence measure. Prediction interval calculations were based on *t*‐distribution.

The Clopper–Pearson method was used to calculate the CI for the proportion calculation of individual studies [42].

## 3 Results

### 3.1 Search and Selection

**FIGURE 1:**
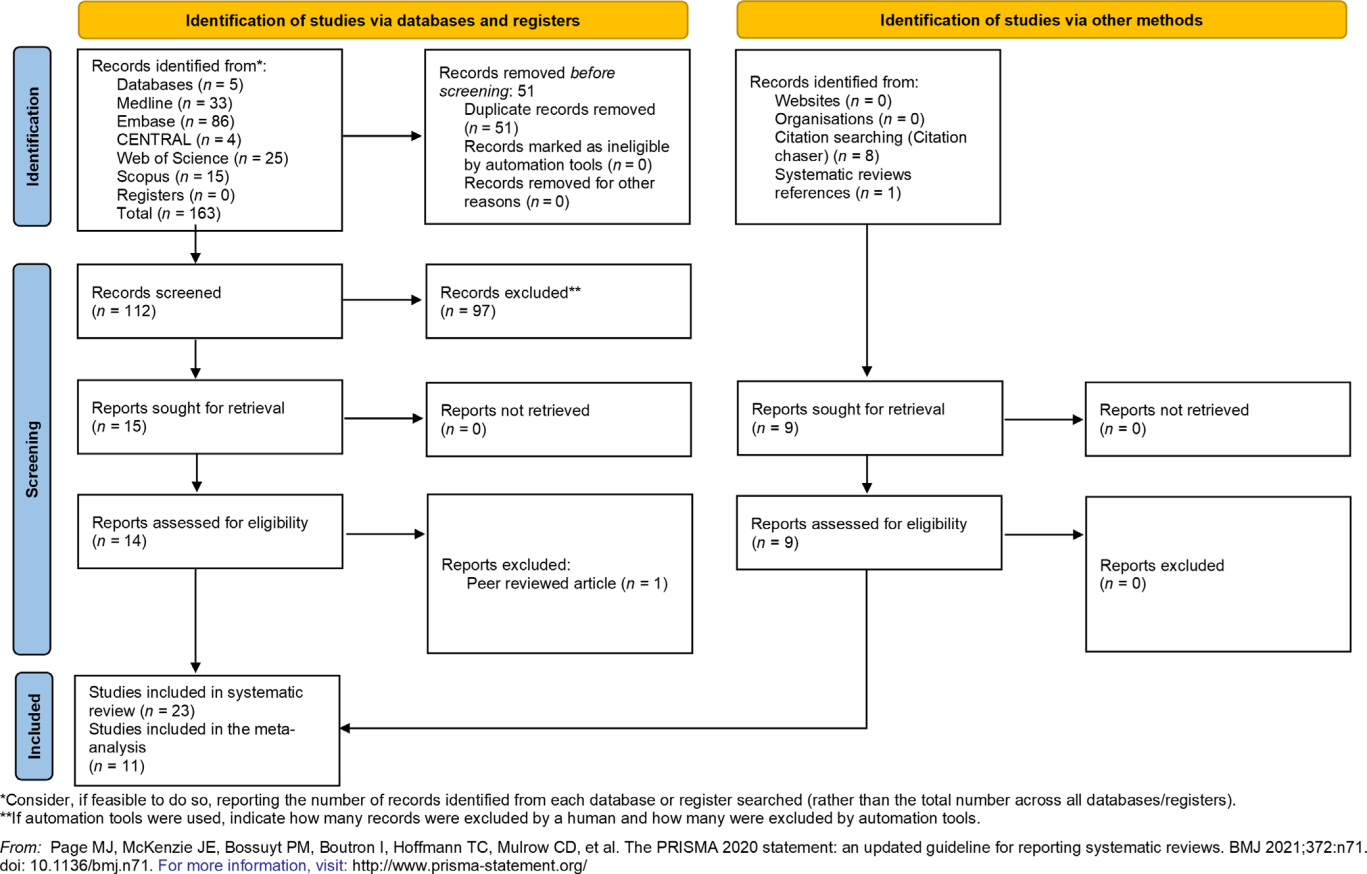
PRISMA 2020 flowchart representing the study selection process.

The search yielded 163 studies. Altogether, 23 studies were retained for data extraction (Figure 1). The agreement between the two investigators for screening titles and abstracts was *κ* = 1, and for full‐text articles, it was also *κ* = 1.

### 3.2 Basic Characteristics of Included Studies

The baseline characteristics of the analyses included are detailed in Table 1. Five different IOSs were investigated in the quantitative analyses: 3Shape TRIOS Color (eight studies) [43, 44, 45, 46, 47, 48, 49, 50], 3Shape TRIOS 3 (14 studies) [6, 19, 20, 51, 52, 53, 54, 55, 56, 57, 58, 59, 60, 61], 3Shape TRIOS 4 (three studies) [57, 58, 59], CEREC Omnicam (six studies) [19, 54, 57, 59, 60, 62], and CEREC Primescan (two studies) [19, 57]. Five different devices were used as comparators: Vita Easyshade SPs 16 times (Vita Easyshade, Vita Easyshade Compact, Vita Easyshade Advance, Vita Easyshade V) [6, 19, 44, 45, 47, 49, 52, 53, 54, 55, 56, 57, 58, 59, 61, 62], SpectroShade SPs four times (SpectroShade, SpectroShade Micro) [20, 43, 45, 50], Rayplicker SP [54], ShadeEye SP once [46], and ColorEye 7000 A colorimeter once each [51]. Of the 23 articles, 18 were in vivo articles where maxillary and mandibular middle incisors, lateral incisors, premolars, or molars of participants were measured [6, 20, 43, 44, 45, 47, 48, 49, 50, 52, 53, 54, 55, 57, 58, 60, 61, 62]. Five articles were in vitro studies [19, 49, 51, 56, 59], whereas, in two studies, VC shade guide tabs [46, 59], in two studies, blocks [19, 51], and in one study, milled restorations [56] were the specimens. In 16 studies, 3D [6, 20, 43, 44, 45, 47, 48, 49, 52, 53, 54, 55, 56, 58, 61, 62], and in 12 studies, VC [6, 19, 20, 46, 47, 50, 53, 57, 58, 59, 60, 62] colors were used. Measurements were taken in cervical, central, and incisal locations in 6 studies [6, 45, 52, 58, 62], in just middle locations in 16 studies [20, 43, 44, 46, 47, 48, 49, 50, 53, 54, 55, 56, 57, 60, 61], and in the center of block in three cases [19, 51, 59]. Accuracy was reported in trueness in 22 studies, where it was expressed in ΔE color difference in six [20, 44, 45, 46, 51, 56], weighted kappa agreement in two studies [43, 47], Cohen's kappa agreement in one article [60], and best match agreement in percentage in 10 studies [6, 19, 20, 49, 55, 57, 58, 59, 61, 62]. Precision was mentioned in 15 articles, where it was expressed in ΔE color difference three times [46, 54, 56], weighted Kappa agreement in one case [43], Fleiss' kappa in two [59, 60], and match agreement in percentage in nine studies [6, 19, 20, 44, 45, 48, 49, 57, 58].

**TABLE 1:** Basic characteristics of studies included.

Reference	Intraoral scanner	Spectrophotometer	Visual method	Sample size	Shade guide code	Type of specimens	Type of experiment	Measured area	Trueness	Precision
Gotfredsen et al., 2015 [43]	3Shape TRIOS Color	SpectroShade	Vita 3D‐Master Vitapan	29 patients (87 teeth), eight patients (24 teeth for repeatability)	Vita 3D‐Master	Maxillary incisors and canine	In vivo	Middle	(TR2‐SS) weighted kappa: 0.8	Value weighted kappa value: 0.8
Brandt et al., 2017 [44]	3Shape TRIOS Color	Vita Easyshade Advanced 4.0	Vita Toothguide 3D‐Master + dentist (V1), Vita Toothguide 3D‐Master + dental technician (V2)	107 patients	Vita 3D‐Master	Maxillary middle incisors	In vivo	Middle	(TR2‐ES) 43.9% ΔE mean = 5.0 ΔE SD = 2.7, (TR2‐V1) 25.2% ΔE mean = 4.0, ΔE SD = 2.7, (TR2‐V2) 33.6% ΔE mean = 3.7, ΔE SD = 3.2; threshold ΔE = 6.8 ACCEPTABLE	TR2 = 78.3%
Mehl et al., 2017 [45]	3Shape TRIOS Color	Vita Easyshade Vita Easyshade Advanced SpectroShade SpectroShade Micro	Vita Toothguide 3D‐Master + 2 dentist (V1), Vita Toothguide 3D‐Master + 2 dental technician (V2)	20 patients (40 teeth)	Vita 3D‐Master	Maxillary middle incisors and canine	In vivo	Cervical, middle, incisial	(TR2‐ES) ΔE mean = 7.0, ΔE SD = 5.0, (TR2‐ESA) ΔE mean = 6.8, ΔE SD = 4.4, (TR2‐SS) ΔE mean = 3.7, ΔE SD = 1.9, (TR2‐SSM) ΔE mean = 3.4, ΔE SD = 2.2 (TR2) relative match = 61.2%	TR2 = 66.7%
Yoon et al., 2018 [46]	3Shape TRIOS Color	ShadeEye	None	Five shade tabs (repetition 10 times on each shade tab)	Vita Classical	Vitapan Classical shade guide	In vitro	Middle	(TR2‐SE) ΔE mean A1 = 7.0, (TR2‐SE) ΔE mean A2 = 13.6, (TR2‐SE) ΔE mean A3 = 12.1, (TR2‐SE) ΔE mean A3.5 = 11.6, (TR2‐SE) ΔE mean A4 = 10.5	(TR2‐SE) ΔE SD A1 = 1.2, (TR2‐SE) ΔE SD A2 = 0.8, (TR2‐SE) ΔE SD A3 = 0.5, (TR2‐SE) ΔE SD A3.5 = 0.7, (TR2‐SE) ΔE SD A4 = 0.5
Culic et al., 2018 [62]	CEREC Omnicam (SW: 4.5.2)	Vita Easyshade Advanced	None	Four patients (80 teeth)	Vita Classical, Vita 3D‐Master	Maxillary and mandibular teeth (10)	In vivo	Cervical, middle, incisial	(TR2‐ESA) VC + 3D = 15%, (TR2‐ESA) VC all = 17.5%, (TR2‐ESA) 3D all = 12.9%, (TR2‐ESA) VC cervical = 21.5%, (TR2‐ESA) 3D cervical = 20%, (TR2‐ESA) VC middle = 22%, (TR2‐ESA) 3D middle = 19%, (TR2‐ESA) VC incisal = 10%, (TR2‐ESA) 3D incisal = 8%	No data
Liberato et al., 2019 [47]	3Shape TRIOS Color	Vita Easyshade Advanced 4.0	Vita Toothguide 3D‐Master (V1), Vita Classical (V2)	28 patients	Vita Classical, Vita 3D‐Master	Maxillary middle incisors	In vivo	Middle	Weighted kappa agreement (TR2) 3D = 0.9, Weighted kappa agreement (TR2) VC = 0.6, Weighted kappa agreement (TR2‐ESA) VC = 0.6, Weighted kappa agreement (TR2‐ESA) 3D = 0.2	No data
Reyes et al., 2019 [48]	3Shape TRIOS Color	None	Vita Toothguide 3D‐Master + 10 prosthodontists	10 patients	Vita 3D‐Master	Maxillary middle incisors	In vivo	Middle	No data	TR2 mean = 86.7%, TR2 SD = 11.5%
Liu et al., 2019 [51]	3Shape TRIOS 3	ColorEye 7000A (colorimeter)	None	120 blocks	None	Color patches, blocks	In vitro	Center	TR3 quadratic polynomial mean ΔE_ab_ = 4.4, TR3 cubic polynomial ΔE_ab_ = 3.8	No data
Yilmaz et al., 2019 [52]	3Shape TRIOS 3	Vita Easyshade Compact	Vita 3D‐Master shade guide	Five patients	Vita 3D‐Master	Maxillary middle incisors	In vivo	Cervical, middle, incisial	(TR3‐ESC) no significant difference	No data
Revilla‐León et al., 2020 [53]	3Shape TRIOS 3	Vita Easyshade V	None	One patient (6 teeth), 10 repetition	Vita Classical, Vita 3D‐Master	Maxillary middle incisor, lateral incisor, canine	In vivo	Middle	(TR3‐ESV) significant difference	No data
Rutkunas et al., 2020 [20]	3Shape TRIOS 3	SpectroShade	None	20 patients (120 teeth)	Vita Classical, Vita 3D‐Master	Maxillary middle incisor, lateral incisor, canine	In vivo	Middle	(TR3‐SS) 3D = 53.3%, (TR3‐SS) VC = 27.5%, (TR3) ΔE 3D < 3.7 = 51.7%, (TR3) ΔE VC < 3.7 = 21.7% NOT AN EXACT MATCH	(TR3) 3D = 90.3%, (TR3) VC = 87.2%
Hampé‐Kautz et al., 2020 [54]	3Shape TRIOS 3 CEREC Omnicam	Vita Easyshade V Rayplicker	Vita 3‐D Master Linearguide + novice practicioner (V1), Vita 3‐D Master Linearguide + expert practicioner (V2)	40 patients	Vita 3D‐Master	Maxillary middle incisor	In vivo	Middle	(IOSs‐SPs) statistically different color	(TR3) ΔE median = 3.4, (Omn) ΔE median = 2.9 (2.45 ΔE acceptability threshold)
Ebeid et al., 2021 [19]	CEREC Omnicam (SW: 4.6.) CEREC Primescan (SW: 5.5.1.) 3Shape TRIOS 3	Vita Easyshade V	None	10 blocks, 10 repetitions per block	Vita Classical	Vita Mark II blocks	In vitro	Center	(TR3‐Block) = 66%, (Pri‐Block) = 63%, (Omn‐Block) = 57%	TR3 = 51.7%, Omn = 51.9%, Pri = 48.4%
Fattouh et al., 2021 [55]	3Shape TRIOS 3	Vita Easyshade Advance	Vita 3D‐Master shade guide (V)	50 patients	Vita 3D‐Master	Maxillary middle incisor	In vivo	Middle	(TR3‐V) agreement = 68%	No data
Czigola et al., 2021 [6]	3Shape TRIOS 3	Vita Easyshade	Vita 3D‐Master Linearguide + dental students (V1), Vita Classical + dental students (V2)	10 patients (3 teeth per patient)	Vita Classical, Vita 3D‐Master	Maxillary middle incisors, premolars, and molars	In vivo	Cervical, middle, incisial	(TR3‐best match) 21.6%	TR3 cervical = 100%, TR3 middle = 75%, TR3 incisal 40%
Antony et al., 2021 [50]	3Shape TRIOS Color	SpectroShade Micro	Vita Classical + clinician (V)	10 patients	Vita Classical	Maxillary middle incisors	In vivo	Middle	(TR2) mean rank = 19.8 IOS is as accurate as the visual method	No data
Sirintawat et al., 2021 [56]	3Shape TRIOS 3 (SW: 1.3.2.0)	Vita Easyshade Advance 5.0	None	Resin model with 30 milled restorations	Vita 3D‐Master	Restoration milled from Vita Mark II blocks	In vitro	Middle	(TR3‐Block) ΔE mean = 6.0, (ΔE threshold: 6.8) statistically significant difference	TR3 ΔE SD = 1.8
Ebeid et al., 2022 [57]	CEREC Omnicam (SW: 4.6.) CEREC Primescan (SW: 5.5.1.) 3Shape TRIOS 3 3Shape TRIOS 4	Vita Easyshade V	Vita Classical + observer (V)	20 patients	Vita Classical	Maxillary middle incisor	In vivo	Middle	(TR‐V) = 75%, (TR4‐V) = 76%, (Omn‐V) = 55%, (Pri‐V) = 69%	TR3 = 79%, TR4‐V = 82%, Omn‐V = 77%, Pri‐V = 82%
Huang et al., 2022 [58]	3Shape TRIOS 3 3Shape TRIOS 4	Vita Easyshade V	Vita 3D‐Master shade guide system + experienced prosthodontist (V1), Vita Classical + experienced prosthodontist (V2)	23 patients (130 teeth)	Vita Classical, Vita 3D‐Master	Maxillary middle incisor	In vivo	Cervical, middle, incisial	(TR3‐ESV) VC = 43%, (TR4‐ESV) VC = 6%, (TR3‐ESV) 3D = 27%, (TR4‐ESV) 3D = 3%.	TR3 VC = 75%, TR4 VC = 72%, TR3 3D = 76%, TR4 3D = 64%
Huang et al., 2023 [59]	CEREC Omnicam (SW: 4.5.2.) 3Shape TRIOS 3 3Shape TRIOS 4	Vita Easyshade V	None	16 shade tabs	Vita Classical	Vita Classical shade guide tabs	In vitro	Center	(TR3‐SG) = 72.5%, (TR4‐SG) = 35%, (Omn‐SG) = 15%	TR3 Fleiss' kappa = 0.9, TR4 Fleiss' kappa = 0.9, Omn Fleiss' kappa = 0.8
Abu‐Hossin et al., 2022 [60]	3Shape TRIOS 3 CEREC Omnicam	None	Vita Classical + experienced dentist (V)	31 patients	Vita Classical	Maxillary middle incisor, canine, and first molar	In vivo	Middle	(TR3‐V) Cohens' kappa = 0.2, (Omn‐V) Cohens' kappa = 0.1	TR3 Fleiss' kappa = 0.6, Omn Fleiss' kappa = 0.5
Vavřičková et al., 2023 [61]	3Shape TRIOS 3	Vita Easyshade Compact Advance	Vita 3D‐Master Vitapan	23 patients	Vita 3D‐Master	Referential tooth	In vivo	Middle	(TR3‐V) = 42.9%	No data
Jagtap et al., 2022 [49]	3Shape TRIOS Color	Vita Easyshade	Vita 3D‐Master Vitapan	20 patients	Vita 3D‐Master	Adjacent tooth	In vivo	Middle	(TR2‐ES) = 70%	TR3 variability = 70%

Abbreviations: ΔE: Color difference; ES: Vita Easyshade; ESA: Vita Easyshade Advance; ESV: Vita Easyshade V; Omn: CEREC Omnicam; Pri: CEREC Primescan; SG: Shade Guide tab; SS: SpectroShade; SSM: SpectroShade Micro; TR2: 3Shape TRIOS Color; TR3: 3Shape TRIOS 3; TR4: 3Shape TRIOS 4; V: visual shade selection.

In total, 11 articles were included in the quantitative analysis [6, 20, 44, 45, 48, 49, 55, 57, 58, 61, 62]. All studies were in vivo articles, where natural teeth were investigated. Seven studies took measurements in the middle third region [20, 44, 48, 49, 55, 57, 61] and four studies published cervical, central, and incisal data [6, 45, 58, 62]. Four main groups were created for the accuracy analyses: trueness in 3D, trueness in VC, precision (repeatability) in 3D, and precision (repeatability) in VC. Data extracted from eligible articles are summarized in Data S3.

### 3.3 Trueness by VITA 3D‐Master

**FIGURE 2:**
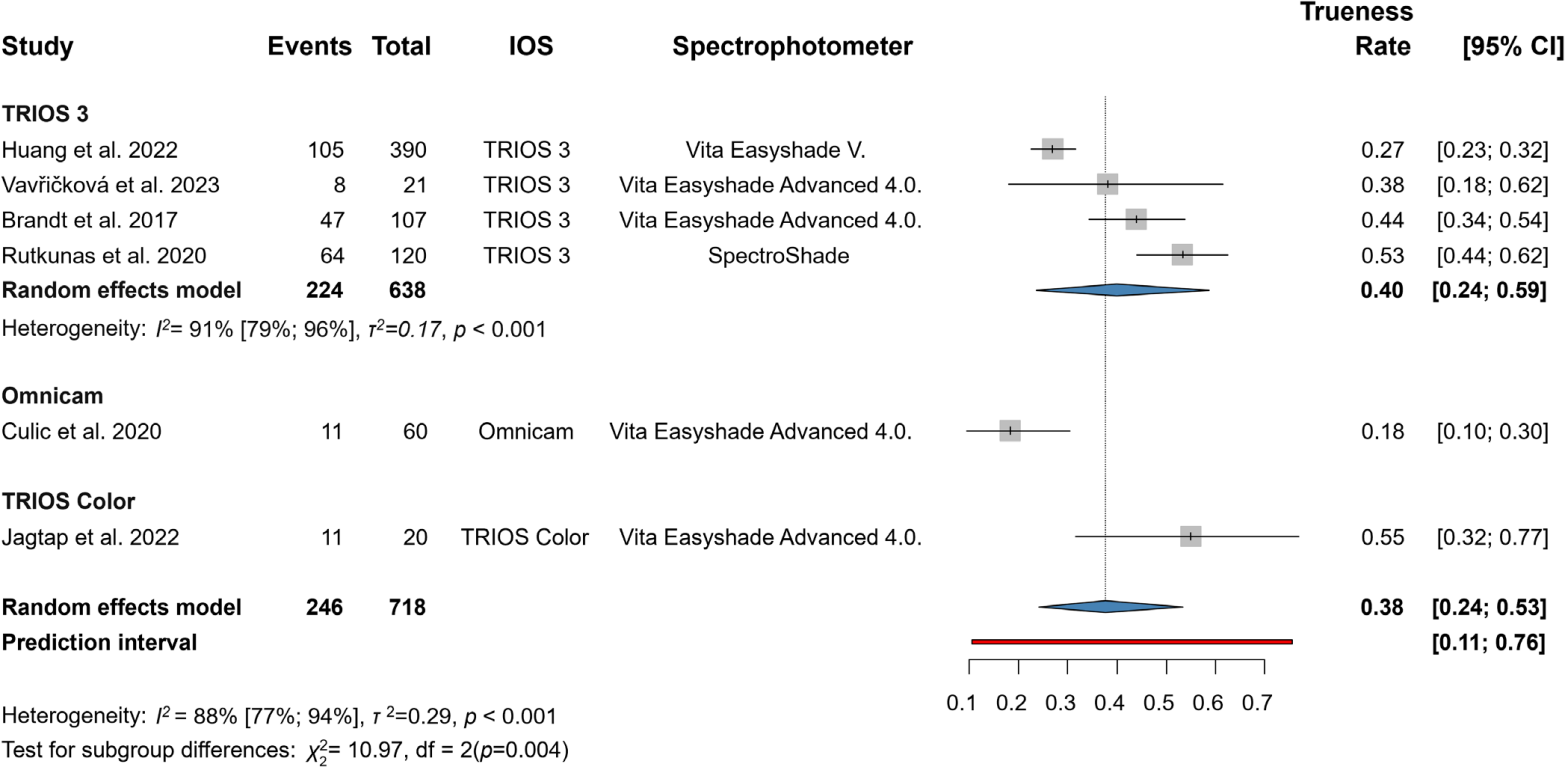
Forest plot representing trueness in VITA 3D‐Master.

Six articles were included in the statistical analyses of the trueness outcome represented by the 3D shade guide system (Figure 2) [20, 44, 49, 58, 61, 62]. The mean trueness of IOSs was clinically significant at 0.38 (CI: 0.24–0.53) and had a statistically significant difference (*p* < 0.001). The mean trueness of 3Shape TRIOS 3 subgroup was 0.4 (CI: 0.24–0.59).

### 3.4 Trueness by VITA Classical

**FIGURE 3:**
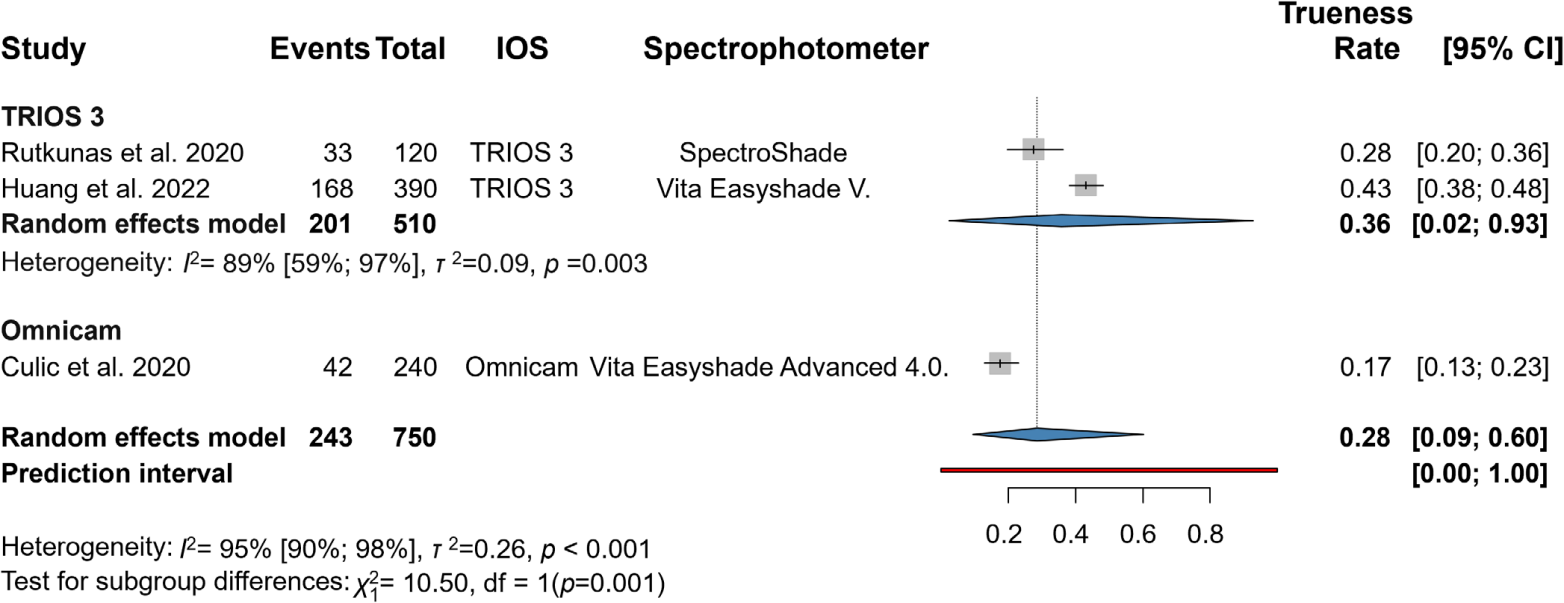
Forest plot representing trueness in VITA Classical.

Three articles were involved in the statistical analyses of the trueness outcome represented by the VC shade guide system (Figure 3) [20, 58, 62]. The mean trueness of IOSs was clinically significant at 0.28 (CI: 0.09–0.60) and had a statistically significant difference (*p* < 0.001).

### 3.5 Precision (Repeatability) by VITA 3D‐Master

#### 3.5.1 Measurement Location: Middle Third

**FIGURE 4:**
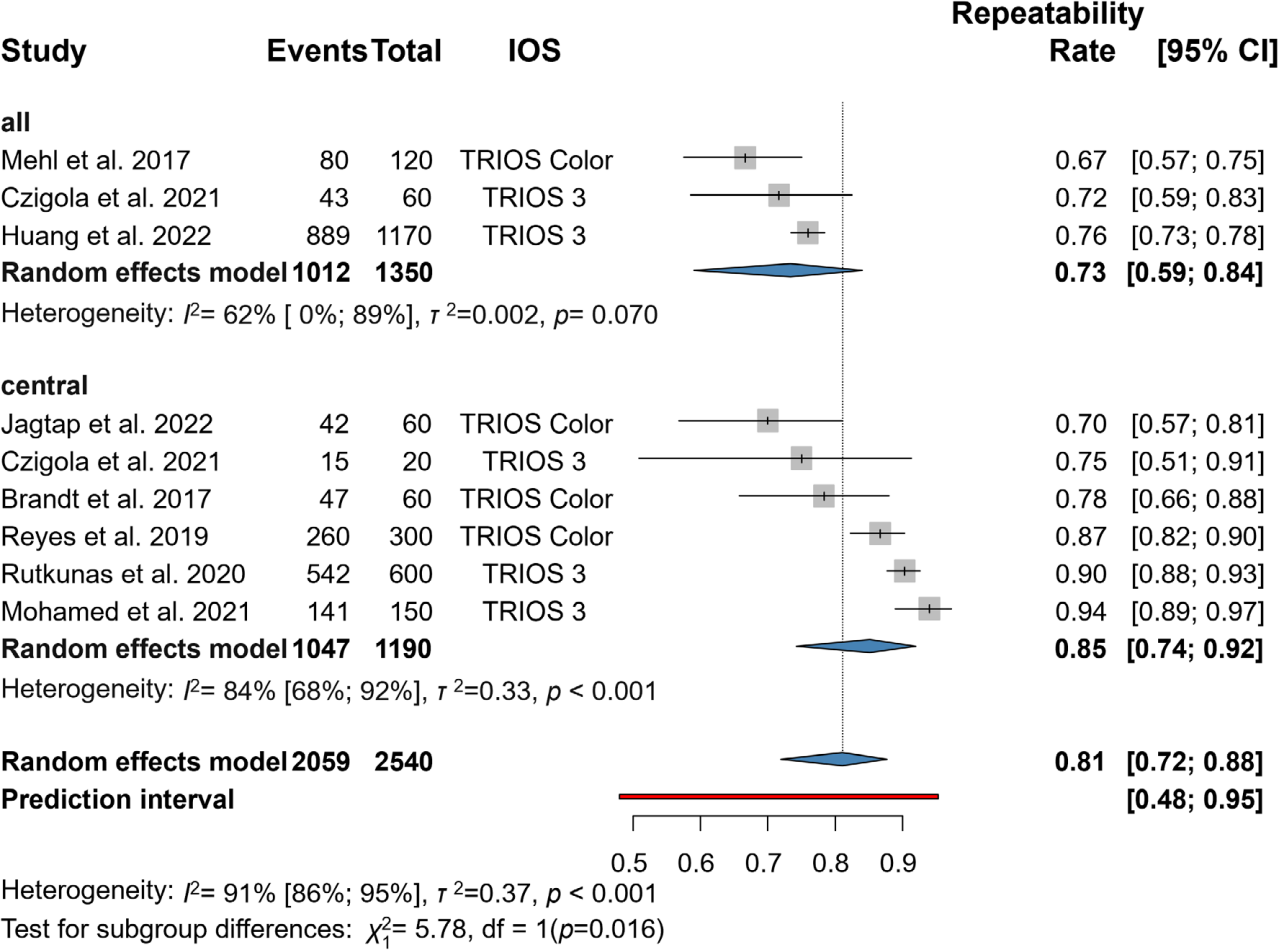
Forest plot representing precision (repeatability) in VITA 3D‐Master with measurement location: Cervical, central, incisal third (all), and central only.

Six articles were involved in the statistical analyses of the repeatability outcome represented by the 3D shade guide system and measured in the middle third location of the reference teeth (Figure 4). The mean trueness of IOSs was high, 0.85 (CI: 0.74–0.92) and had a statistically significant difference (*p* < 0.001).

#### 3.5.2 Measurement Location: Cervical, Central, and Incisal Third

Three articles were included in the statistical analyses of the repeatability outcome represented by the 3D shade guide system and measured in the middle third location of the reference teeth (Figure 4) [6, 45, 58]. The mean trueness of IOSs was clinically acceptable, 0.73 (CI: 0.59–0.84), and had a statistically insignificant difference (*p* < 0.070).

### 3.6 Precision (Repeatability) by VITA Classical

**FIGURE 5:**
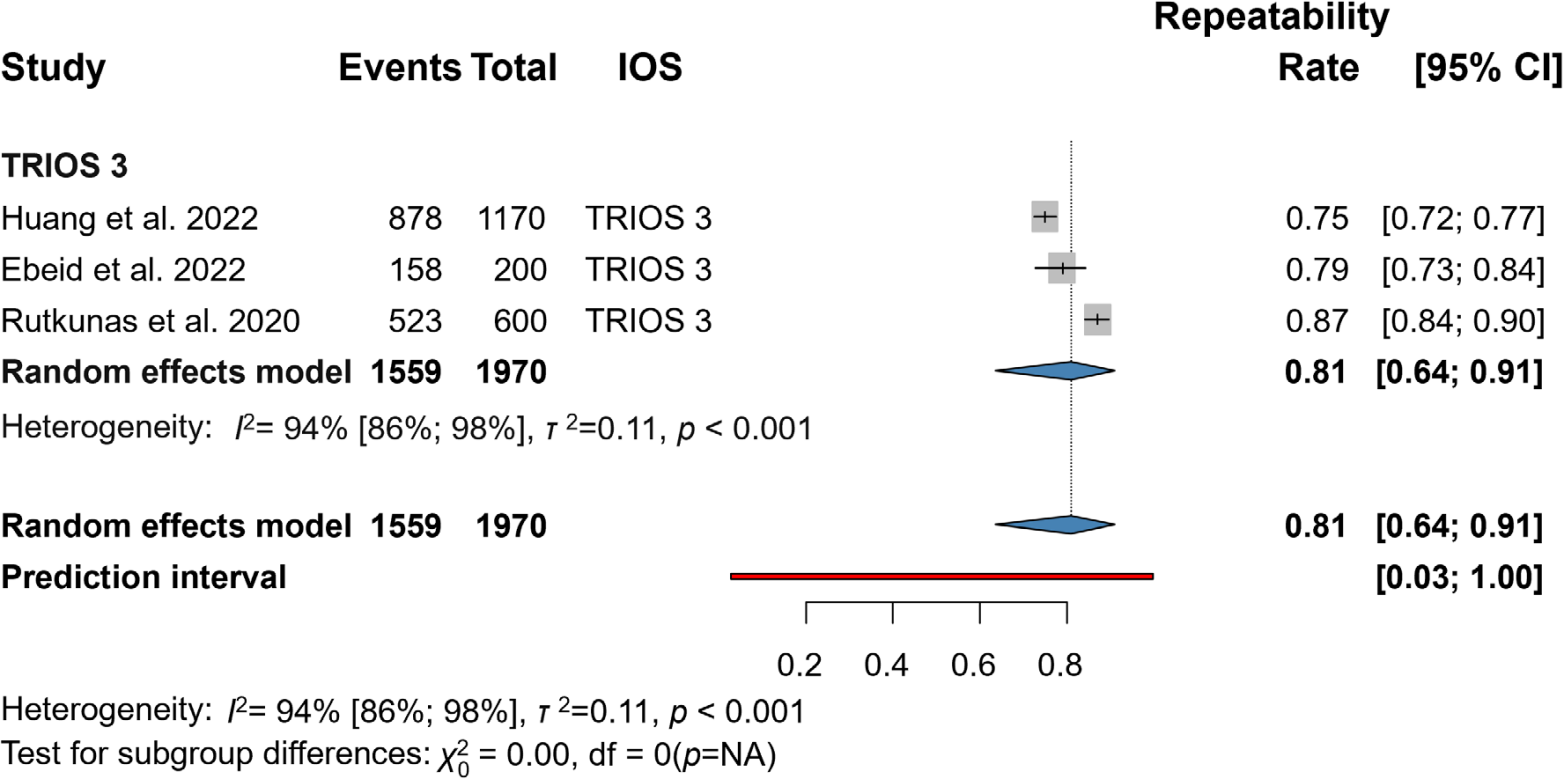
Forest plot representing precision (repeatability) in VITA Classical.

Three articles were included in the statistical analyses of the repeatability outcome represented by the VC system with 3Shape TRIOS 3 IOS (Figure 5) [20, 57, 58]. The mean trueness of IOSs was clinically acceptable, 0.81 (CI: 0.64–0.91), and had a statistically significant difference (*p* < 0.001).

### 3.7 Risk of Bias Assessment

The overall risk for one systematic review on the topic was low at 100% [31], and 82% in another study [22]; for our study was 78% (Table 2). Therefore, the overall results were similar in all studies, showing a low risk of bias. (Data S4) [6, 19, 20, 43, 44, 45, 46, 47, 48, 49, 50, 51, 52, 53, 54, 55, 56, 57, 58, 59, 60, 61, 62].

**TABLE 2:** Risk of bias assessment using the JBI checklist.

Reference	Risk of Bias									Overall
	Q1	Q2	Q3	Q4	Q5	Q6	Q7	Q8	Q9	
Gotfredsen et al., 2015 [43]	Low	Low	Low	Low	Unclear	Low	Low	Unclear	Low	Low
Brandt et al., 2017 [44]	Low	Low	Unclear	Low	High	High	High	High	Low	Unclear
Mehl et al., 2017 [45]	Low	High	Unclear	High	Low	High	High	Low	Low	Unclear
Yoon et al., 2018 [46]	Low	Low	High	Low	Low	High	Low	Low	Low	Low
Culic et al., 2018 [62]	Low	Low	Unclear	Low	High	High	Low	High	Unclear	Low
Liberato et al., 2019 [47]	Low	Low	Unclear	Low	Low	High	Low	Unclear	Low	Low
Reyes et al., 2019 [48]	Low	Low	Low	Low	Low	Low	Low	Low	Low	Low
Liu et al., 2019 [51]	Low	High	Unclear	Low	Unclear	High	Low	Unclear	Low	Low
Yilmaz et al., 2019 [52]	Low	Low	Low	Low	High	High	Low	Low	Low	Low
Revilla‐León et al., 2020 [53]	Low	Low	Low	Low	Low	High	Low	Low	Low	Low
Rutkūnas et al., 2020 [20]	Low	Low	Low	Low	Low	High	Low	Low	Low	Low
Hampé‐Kautz et al., 2020 [54]	Low	Low	Unclear	Low	High	High	High	Unclear	High	High
Ebeid et al., 2021 [19]	Low	Low	Low	Low	Low	High	Low	Low	Low	Low
Fattouh et al., 2021 [55]	Low	Low	Low	Low	Unclear	High	High	Unclear	High	Low
Czigola et al., 2021 [6]	Low	Low	Low	Low	Low	Low	Low	Low	Low	Low
Sirintawat et al., 2021 [56]	Low	Low	Low	Low	Low	High	High	Low	Low	Low
Antony et al., 2021 [50]	Low	Low	Unclear	Low	High	High	Low	High	High	High
Ebeid et al., 2022 [57]	Low	Low	Low	High	Low	High	Low	Low	Low	Low
Huang et al., 2022 (1) [58]	Low	Low	Low	Low	Low	High	Low	Low	Low	Low
Huang et al., 2022 (2) [59]	Low	Low	Low	Low	High	High	Low	High	Low	Low
Abu‐Hossin et al., 2022 [60]	Low	Low	Low	High	Low	Low	Low	Low	Low	Low
Jagtap et al., 2022 [49]	Low	Low	Unclear	Low	High	High	High	High	High	High
Vavřičková et al., 2023 [61]	Low	Low	Low	Low	High	High	High	Unclear	Low	Low

### 3.8 Publication Bias and Heterogeneity

Publication bias was assessed using Peters (modified Egger's) test. The results for publication bias in different subgroups are presented in Data S5. Among the subgroups, heterogeneity was between 62% and 94%.

### 3.9 Certainty Assessment

The results of the certainty assessment with GRADE Pro are presented in Data S6. Very low certainty of evidence was found in the accuracy groups of critical importance due to different IOS systems and different types of SPs used. Furthermore, SPs used as gold standards do not detect tooth color with 100% accuracy, so there is no absolute proof of the original tooth color. The certainty of the evidence in the repeatability groups was moderate, which is critical due to the different IOS systems used.

## 4 Discussion

The research hypothesis was rejected as significant differences in shade matching were found between IOSs and SPs. The shade‐matching accuracy of IOSs was assessed separately for trueness and precision.

An IOS emits light onto the area to be scanned; sensors capture the reflected light, which the scanner software processes to create an image of the scanned region. Some scanners use photo imaging methods such as 3Shape Trios (3Shape, Copenhagen, Denmark), and others utilize video imaging methods such as Omnicam and Primescan (Dentsply Sirona, Bensheim, Germany) [63]. Optical sectioning, a technique from confocal laser scanning microscopy, is used by 3Shape Trios to obtain sharp images at specific depths. A computer obtains and reconstructs point‐by‐point images. In active triangulation methods, a light beam is projected onto the scene using technology similar to Omnicam and Primescan image acquisition. The position of the target object is then determined by capturing the reflection of the light beam. IOSs can distinguish between soft and hard tissue features by clarity and color in their images [19]. Next, the software allows the shade of the selected tooth location to be determined using VC and 3D shade guidelines. A limitation of IOS software is that it cannot show CIELab/CIELch values; therefore, color difference values cannot be generated without a conversion chart.

In contrast, SPs can determine the color of an object by evaluating its transmittance or spectral reflectance curve. The white light source in a SP is a LED lamp or tungsten filament bulb that emits light at a wavelength of 400–700 nm. When the light hits the item, a prism lowers its wavelength to bands between 10 and 20 nm, from which it can be reflected, transmitted, or scattered. The amount of light is measured that the object emits or transmits across every visible wavelength band. Some dental SPs can use a mouthpiece to separate the oral cavity from outside light, removing the effect of ambient light on color readings [16, 44]. SPs can measure the entire surface or a specific area of a tooth while displaying variations in lighting, sensors, filters, and angle of irradiance/reflection [64, 65]. Color measurements are typically translated into an equivalent shade tab. When VITA 3D‐Master and VC are used, the Easyshade SP calculates the tooth shade using CIELab/CIELCh values. The manufacturer claims that the 20 W halogen bulb provides D65 illumination. Five generations have been released so far. The most recent version is VITA Easyshade V, whereas the advanced versions are VITA Easyshade Compact and Advance 4.0. Spectrophotometry based on LED technology is combined with digital color imaging in SpectroShade Micro. A linear polarized filter helps this instrument by preventing gloss‐induced reflections. The device memory stores various shade guide data, allowing for comparing images obtained with various shades [9].

**FIGURE 6:**
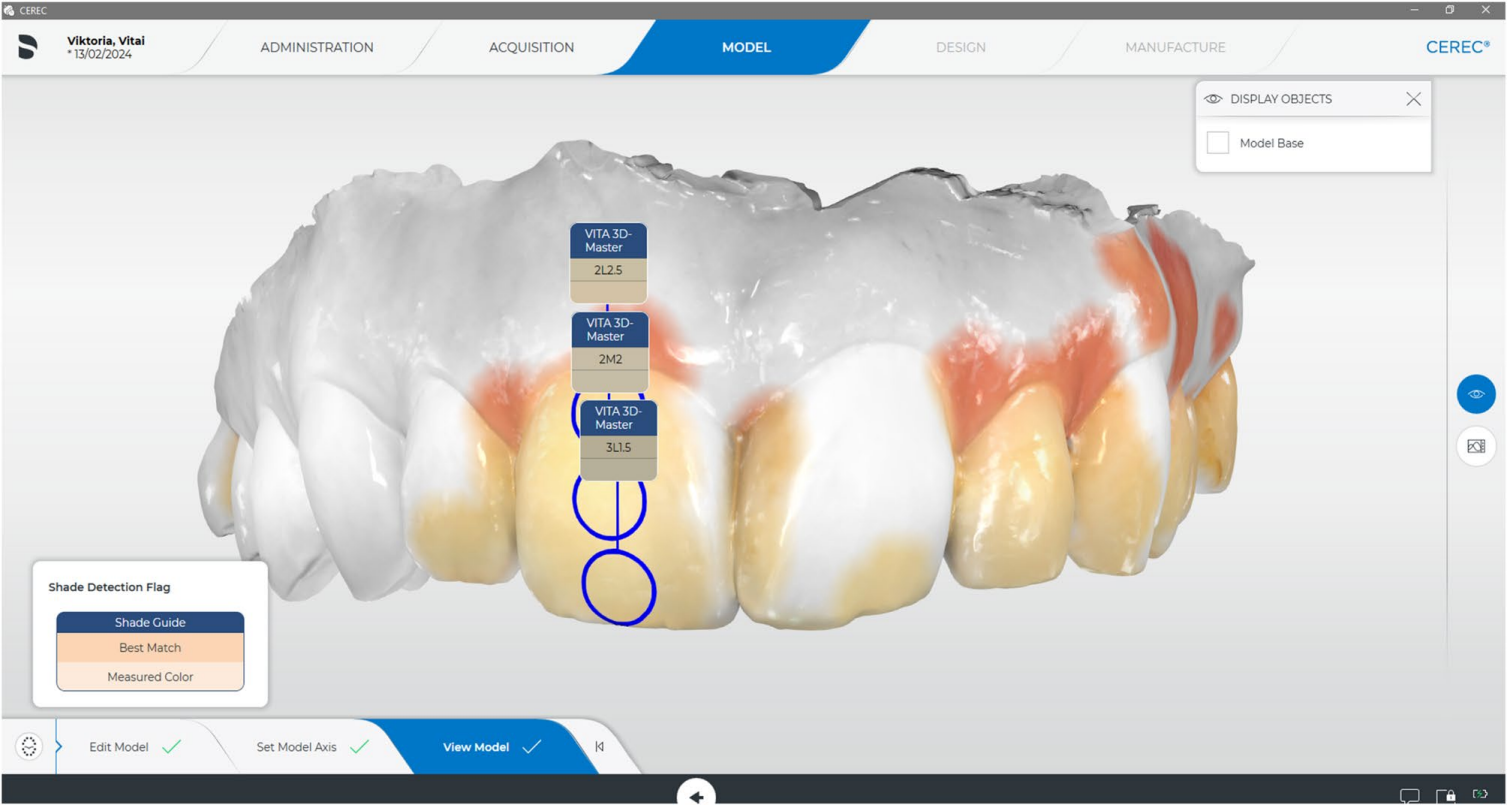
CEREC Primescan software with the shade selection tool. The incisal shade looks darker than the central and cervical locations.

The translucency of teeth increases from the dental cervix to the incisal part, causing light to be transmitted more easily. Unfortunately, the IOS can detect a darker background (oral cavity) in the incisal region, causing a shade darker than the true shade, which can also be detected in the software of IOS, where we can see a dark incisal edge followed by a lighter middle and neck region (Figure 6).

In the literature, SPs demonstrate better accuracy compared to other shade‐taking instruments [66, 67]. They are said to be the most appropriate tooth‐color measurements, with over 96% reliability [17, 66, 68]. In a study, Easyshade and SpectroShade Micro produced higher b* and higher a* values, respectively. This could be attributed to their different optical geometries and the lower irradiation area of Easyshade, which may impact CIELab values. According to a study [69, 70], Easyshade Advance 4.0 and V were equivalent in terms of color measurements for premolars and incisors and were also accurate and precise. Although SpectroShade is more accurate than Easyshade in vivo, the trueness and precision of Easyshade may be significantly impacted in freehand situations [68]. Although the first iteration of Easyshade demonstrated inferior accuracy compared to a reference device (ΔE_ab_ = 13.4–15.4) [71], based on a systematic review, Easyshade gives excellent accuracy and precision, outperforming the photo colorimetric approach [72]. SpectroShade Micro has demonstrated accurate color measurements with systematic and random errors below the PT (ΔE_ab_ < 1) in an in vivo investigation [73]. SPs are able to detect minor color variations as some manufacturers report their detection limit for in vitro quantification of monochromatic objects to be ΔΕ_ab_ = 0.1 [9].

This MA showed a wide range of precision in the involved articles that published data on the SP used. Low precision (32%) was found when dental students used Vita Easyshade in an in vivo study [6] and when blocks were measured in vitro circumstances (44.3%) [19]. Higher precision results were found in the in vivo studies with expert operators using Vita Easyshade ranging from 68.3% to 93.5% [20, 44, 45, 57]. SpectroShade SPs had similar results, with 61.7% and 71.7%, respectively [45].

Our findings indicate that IOSs have a clinically acceptable high repeatability of 81%, comparable to the precision of SPs; nevertheless, they are not clinically acceptable due to their poor trueness (28%–38%). Therefore, high precision is useless if the trueness is low because we can repeatedly measure the incorrect shade. Similarly, the information accuracy of RGB devices is questionable, including photo‐taking shade matching, as they define the color proprieties of a captured image rather than measure the instrument reading [72, 74]. This type of systematic error may be resolved using new software algorithms.

When we tried to replicate the measured shade, we ran into a further problem. Unfortunately, not all dental materials work with the 3D or VC [9]. Significant variances were discovered when shade guides and various material block colors were compared [75]. Therefore, replicating true tooth color can still be difficult even if we can identify the correct color in the shade guidance system.

### 4.1 Strengths and Limitations

Regarding the strengths of our analysis, we followed our preregistered protocol. For the quantitative synthesis, we used only in vivo articles. Heterogeneity was reduced as much as possible by subgrouping data. The outcomes investigated were of critical importance, and the overall risk of bias was low at 78%.

Considering the limitations of this work, we found that heterogeneity among the studies was high in all groups due to different original tooth shades of the reference, location of measurements, type of shade guide system used, IOS types, SP types, different operators, different light conditions, and different measurement setups.

The reference tooth shade may affect trueness and repeatability. In the articles, maxillary central incisors were the most investigated subjects, but all types of teeth, such as lateral incisors, canines, premolars, and molars, were selected for shade selection. Molars and premolars are known to have a darker shade than central incisors [76]. IOS devices can measure darker shades more precisely than lighter ones, although this claim has not been verified [46].

Different measurement locations, such as cervical, central, and incisal regions, can greatly affect the accuracy and repeatability of IOSs due to different levels of translucency [6]. A similar problem is encountered in contact‐type SPs: the difference in tooth color and the thickness of the labial enamel have a weak‐to‐moderate positive association. The color of the teeth is more greatly affected by thicker labial enamel [77].

A number of studies have searched for differences between VC and 3D shade guide shade matching when using IOSs. A systematic review found that colors measured in 3D shades had better accuracy than in VC [31].

Although the basics of shade measuring principles are the same in the IOS software, there can be differences between the devices [78]. This can be due to the shade‐calculating algorithm or hardware differences, but our study does not include these causative effects. Furthermore, not all IOSs capable of measuring tooth shade have been investigated [79].

Although SPs are considered a good option for reference, there can be differences between them, and several factors can affect their accuracy. The main variables that can affect spectrophotometric measurements include the size of the surface measured, proper positioning of the probe, angulation, and centering, the color analysis software of the device, and the shade guide mode [9, 21].

Operators can affect accuracy. Articles discuss different levels of experience, such as experienced and inexperienced. Sometimes, more than one operator was involved in the study, where inter‐rater reliability was useful information to describe experimenter bias [47].

As with other optical devices, IOSs are also sensitive to light conditions. A study found that the best results were detected under 0 lx conditions with no light [53]. In most articles, measured lux data were not available, and measurements were performed under daylight conditions in a room [60]. As the timing and location of the studies differed, this can cause a great discrepancy in lux change. However, color temperature has not yet been proven to impact accuracy [52].

Another factor that can cause great heterogeneity is the different measurement setups. The number of measurement repetitions varied in trueness and repeatability studies. Articles without a calculated sample size involve a great risk of bias.

### 4.2 Implications for Practice and Research

Translating scientific knowledge into everyday practice has crucial importance [80, 81]. Based on our results, we recommend against using IOSs for tooth shade determination because there is no evidence that they can be a reliable solution in everyday dental practice. As we lack information on all IOSs, further data collection is needed to investigate other available devices.

It may be useful to consider the translucency error and how the background—black, gray, and white—affects the accuracy of IOSs.

Novel studies should consider using more coherent outcomes, as different measurement methods were used in the articles, which were not always comparable. Important outcomes such as ΔE_00_ calculated with the CIEDE2000 equation are important to investigate in future studies. With the ΔE_00_, color differences can be more accurately represented, and the perceptibility and ATs can be used to assess clinical significance. Using coherent converting charts when calculating color difference is important, as IOS software does not show lab values.

In future studies, it is imperative to recognize that the accuracy of measurements may be affected by the specific location where they are taken. Therefore, for further systematic comparisons, it is recommended that data from multiple locations be measured and published.

As in clinical studies, the original color of the tooth is unknown; it will be interesting to investigate the accuracy of IOS compared to other references, such as visual methods. Using the same study setup as was used to establish perceptibility and ATs may also be a good option [26]. IOS accuracy data from different references can be the base of a network analysis that can provide more reliable data on the accuracy of IOS in tooth shade determination.

## 5 Conclusion

The low trueness made the accuracy of IOSs unacceptable compared to SPs.

In conclusion, on the basis of systematically investigated scientific articles, the use of IOSs for tooth shade selection is currently not recommended.

## Author Contributions

**Viktória Vitai:** conceptualization, methodology, investigation, data curation, writing – original draft, visualization, project administration. **Anna Németh:** investigation, validation, writing – review and editing. **Brigitta Teutsch:** conceptualization, methodology, supervision, project administration, writing – review and editing. **Kata Kelemen:** conceptualization, methodology, supervision, project administration, writing – review and editing. **Alíz Fazekas:** formal analysis, data curation, writing – review and editing. **Péter Hegyi:** conceptualization, methodology, writing – review and editing, supervision. **Orsolya Németh:** writing – review and editing, supervision. **Beáta Kerémi:** writing – review and editing, supervision. **Judit Borbély:** conceptualization, methodology, validation, project administration, writing – review and editing, supervision. All authors certify that they have participated sufficiently in the work to take public responsibility for the content, including participation in the concept, design, analysis, writing, or revision of the manuscript.

## Ethics Statement

No ethical approval was required for this systematic review with meta‐analysis, as all data were already published in peer‐reviewed journals. No patients were involved in the design, conduct, or interpretation of our study. The datasets used in this study can be found in the full‐text articles included in the systematic review and meta‐analysis.

## Conflicts of Interest

The authors declare no conflicts of interest.

## Supporting information


**Data S1.** PRISMA 2020 Checklist.

**Data S2.** Search key.

**Data S3.** Extracted data from studies included.

**Data S4.** Risk of bias assessment using the JBI checklist.

**Data S5.** Publication bias in different subgroups.

**Data S6.** Results of the certainty assessment using GRADE Pro.

## Data Availability

The data that supports the findings of this study are available in the supplementary material of this article.
