# Supplementary material for: Color Comparison Between Intraoral Scanner and Spectrophotometer Shade Matching: A Systematic Review and Meta‐Analysis
Source: J Esthet Restor Dent. 2024 Sep 9;37(2):361–77. doi: 10.1111/jerd.13309 (PMC11927804; doi:10.1111/jerd.13309)
Supplement: Supplementary file 2 — Data S2. Search key. [file JERD-37-361-s004.docx]

**Supplementary Material 2.**

**Search key:**

*(shade determination OR tooth colour OR tooth color OR tooth shade OR natural tooth shade OR shade selection) AND* *(intraoral scanner OR intraoral-scanner OR IOS OR intraoral scan) AND (spectrophotometer OR instrument OR Easyshade OR SpectroShade OR colorimeter)*
