# Supplementary material for: Color Comparison Between Intraoral Scanner and Spectrophotometer Shade Matching: A Systematic Review and Meta‐Analysis
Source: J Esthet Restor Dent. 2024 Sep 9;37(2):361–77. doi: 10.1111/jerd.13309 (PMC11927804; doi:10.1111/jerd.13309)
Supplement: Supplementary file 5 — Data S5. Publication bias in different subgroups. [file JERD-37-361-s001.docx]

**The results of publication bias in different subgroups**

**Funnel plot of subgroup Accuracy 3D**

**
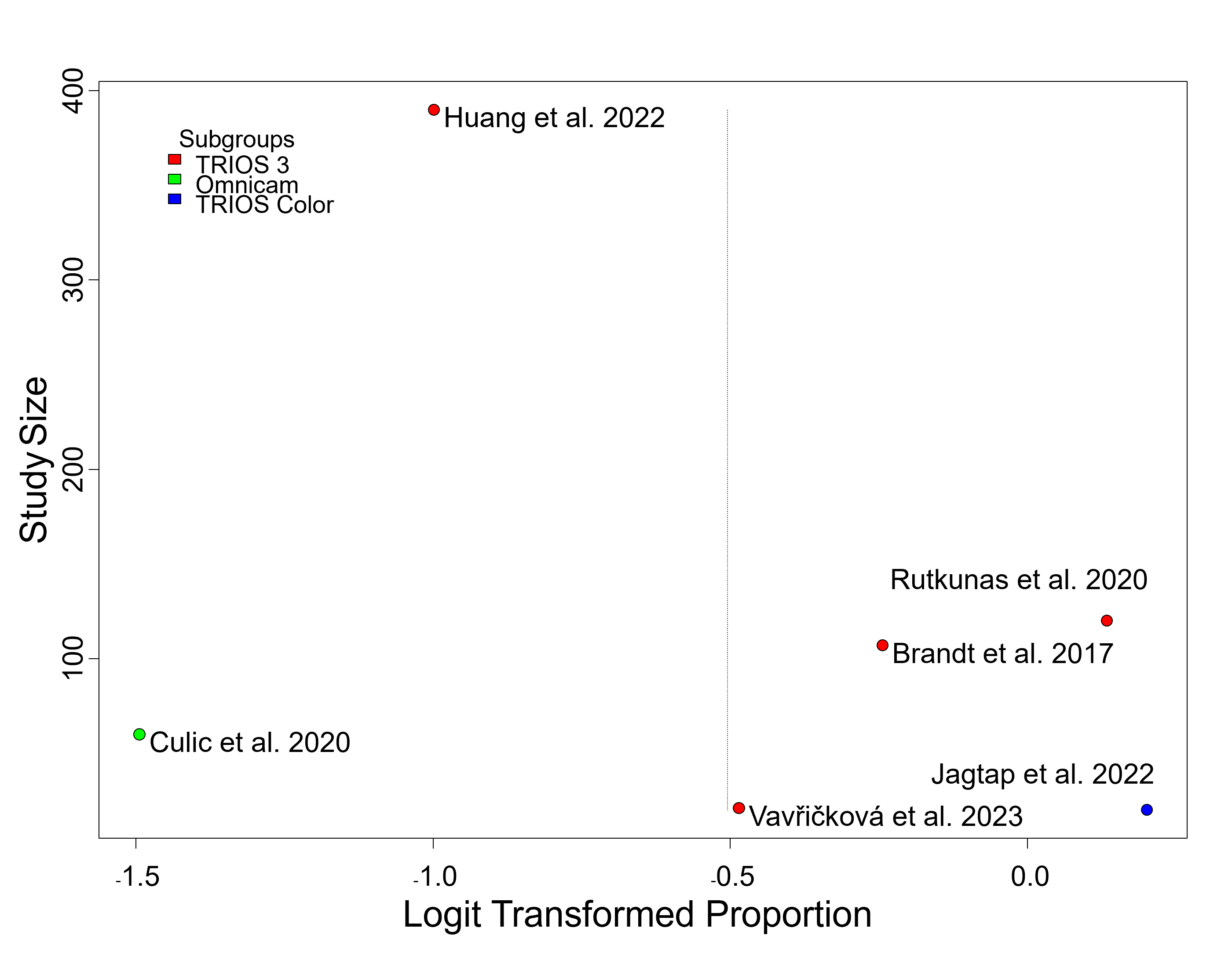
**

**Funnel plot of subgroup Accuracy VC**

**
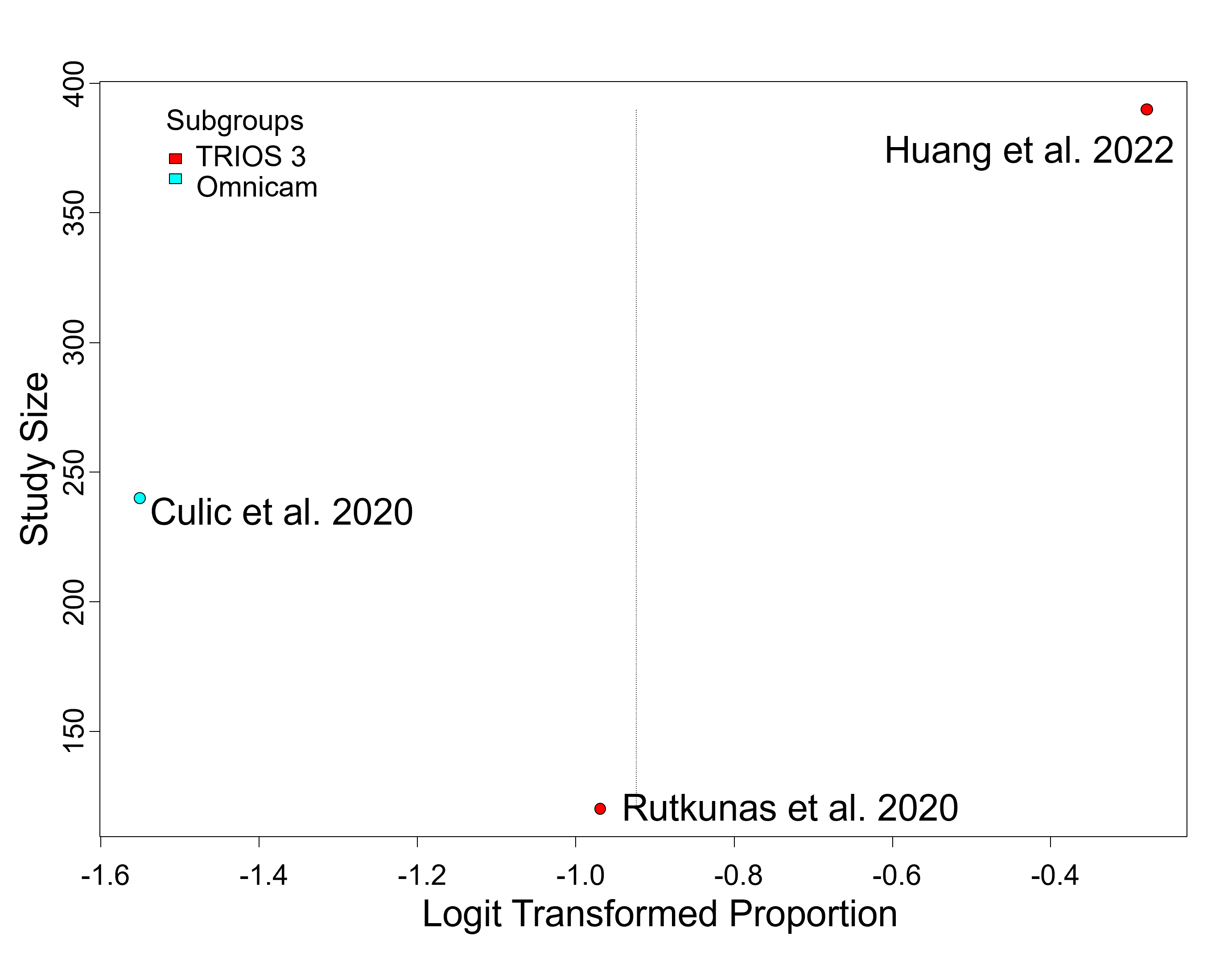
**

**Repeatability 3D**

**
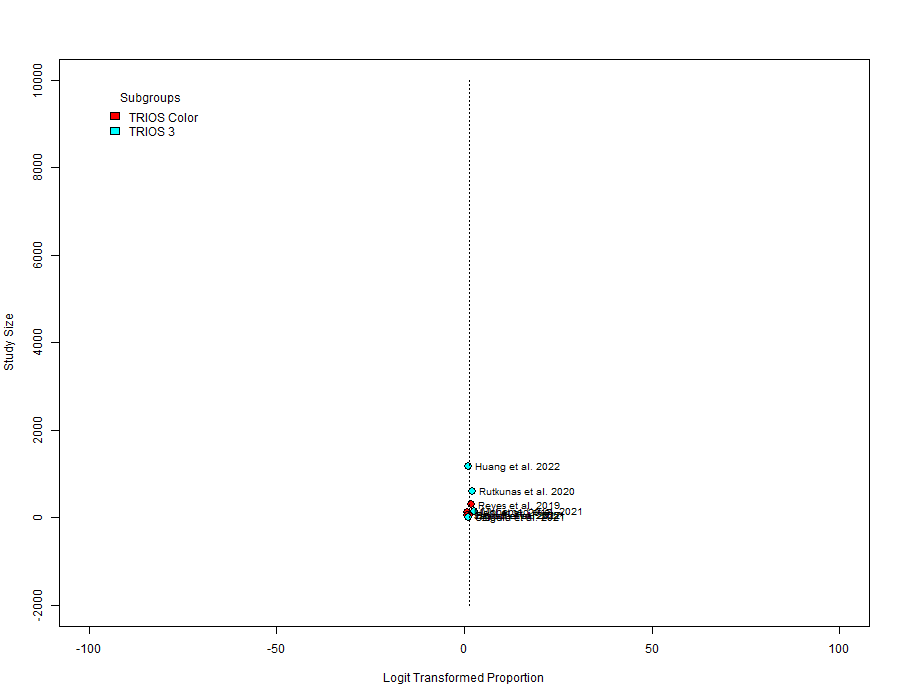
**

**Repeatability 3D
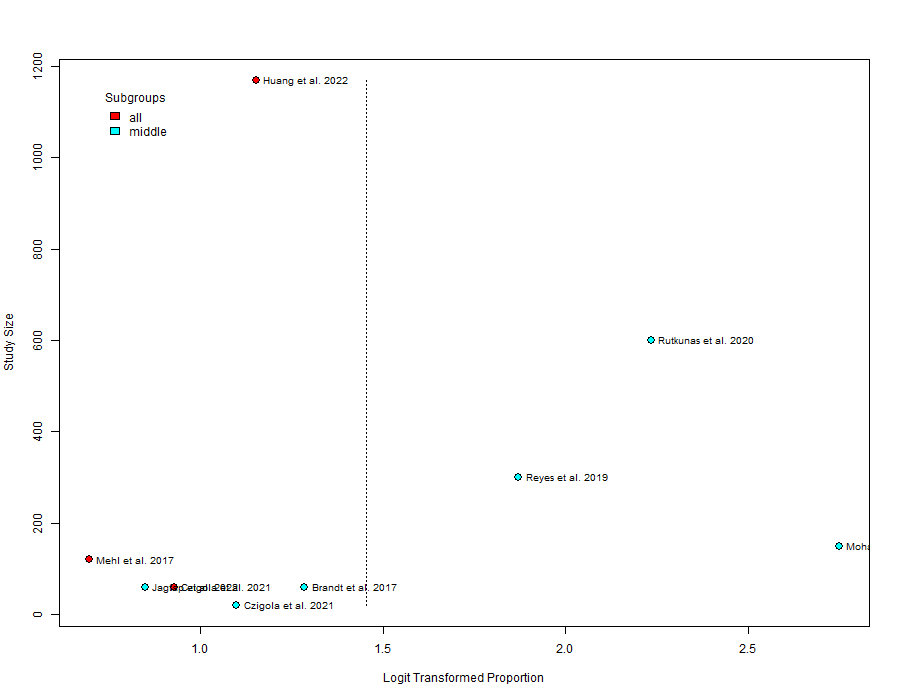
**
