# Supplementary material for: Color Comparison Between Intraoral Scanner and Spectrophotometer Shade Matching: A Systematic Review and Meta‐Analysis
Source: J Esthet Restor Dent. 2024 Sep 9;37(2):361–77. doi: 10.1111/jerd.13309 (PMC11927804; doi:10.1111/jerd.13309)
Supplement: Supplementary file 6 — Data S6. Results of the certainty assessment using GRADE Pro. [file JERD-37-361-s003.docx]

**Supplementary material: Certainty Assessment with GRADE Pro**

| **Certainty assessment** | | | | | | | **№ of patients** | | **Effect** | | **Certainty** | **Importance** |
| --- | --- | --- | --- | --- | --- | --- | --- | --- | --- | --- | --- | --- |
| **№ of studies** | **Study design** | **Risk of bias** | **Inconsistency** | **Indirectness** | **Imprecision** | **Other considerations** | **intraoral scanner** | **spectrophotometer** | **Relative (95% CI)** | **Absolute (95% CI)** |  |  |
| **Percentage of correct match (trueness) in VC (follow-up: mean 1 weeks; assessed with: incidence; Scale from: 0 to 100)** | | | | | | | | | | | | |
| 5 | observational studies | not serious | serious^a^ | serious^b^ | not serious | strong association | 718 | 718 | - | 0 **100 % higher** (0 to 1 higher) | ⨁◯◯◯ Very low | CRITICAL |
| **Percentage of correct match (trueness) in 3D (follow-up: mean 1 weeks; assessed with: percentage; Scale from: 0 to 100)** | | | | | | | | | | | | |
| 3 | observational studies | not serious^a^ | serious^b^ | serious^b^ | not serious | strong association | 750 | 750 | - | 0 **100 % higher** (0 to 1 higher) | ⨁◯◯◯ Very low | CRITICAL |
| **Percentage of the same match (precision) in VC (follow-up: mean 1 weeks; assessed with: percentage; Scale from: 0 to 100)** | | | | | | | | | | | | |
| 3 | observational studies | not serious | not serious | not serious | not serious | strong association | 1970 |  | - | 0 **100 % higher** (0 to 1 higher) | ⨁⨁⨁◯ Moderate | CRITICAL |
| **Percentage of the same match (precision) in 3D (follow-up: mean 1 weeks; assessed with: percentage; Scale from: 0 to 100)** | | | | | | | | | | | | |
| 8 | observational studies | not serious | not serious | not serious | not serious | strong association | 2520 |  | - | 0 **100 % higher** (0 to 1 higher) | ⨁⨁⨁◯ Moderate | CRITICAL |

**CI:** confidence interval

#### Explanations

a. In the studies different IOSs systems and different types of spectrophotometers were used.

b. Even spectrophotometers, which can be used as gold standards, are not 100% accurate in detecting the correct tooth color, so there is no absolute proof of the original color of the teeth.
